# Children’s Improvement After Language and Rhythm Training With the Digital Medical Device Poppins for Dyslexia: Single-Arm Intervention Study

**DOI:** 10.2196/76435

**Published:** 2025-08-01

**Authors:** Charline Grossard, Melanie Descamps, Hugues Pellerin, François Vonthron, David Cohen

**Affiliations:** 1 Department of Child and Adolescent Psychiatry Pitié-Salpêtrière Hospital Paris France; 2 Poppins Paris France

**Keywords:** serious game, medical device, specific learning disorder, reading, rhythm

## Abstract

**Background:**

Specific learning disorder in reading (SLD reading), commonly named dyslexia, is a neurodevelopmental condition affecting reading. Current best practice recommendations for SLD reading emphasize the necessity of including graphophonological interventions. The serious game Mila-Learn, which is based on rhythm training, showed promising results in a prior randomized trial. However, it lacked a component of graphophonological training.

**Objective:**

This study aimed to evaluate the effectiveness of Poppins, a new digital medical device that combines rhythm-based and graphophonological training for improving reading and phonological skills in children with SLD reading. We also explored its performance against Mila-Learn, the earlier version based on rhythm training only.

**Methods:**

A single-arm study without an active control group was conducted with 38 children (aged 7-11 years) diagnosed with SLD reading. The participants completed an 8-week training program with Poppins (five 20-minute sessions per week). Pre- and posttraining assessments measured reading accuracy and speed, phoneme deletion, and phonological discrimination. Statistical analysis included pre- and postcomparisons (primary analysis) and comparisons with children's improvement from a previous randomized controlled trial of Mila-Learn, an earlier version of the device (exploratory analysis).

**Results:**

The participants demonstrated significant improvements in reading accuracy (+11.46 words correctly read; *P*<.001), reading speed (+10.26 words read; *P*<.001), and phoneme deletion (+2.87 points; *P*<.001). No significant change was observed in reading comprehension for younger participants (grades 2-3; *P*=.09), although improvements were noted in older children (grades 4-5, *P*=.03). Exploratory analysis comparing children’s improvements with Mila-Learn and Poppins revealed similar gains in reading accuracy and speed but revealed superior improvement in phonological skills for the Poppins group, with a moderate effect size according to the benchmarks by Cohen (Cohen *d*=0.48, *P*=.02).

**Conclusions:**

Poppins is an effective and safe tool for enhancing reading and phonological skills in children with SLD reading. By integrating rhythm-based and graphophonological exercises, the device aligns with best practice recommendations for curative intervention. Future research should explore its long-term effects and medicoeconomic impact and compare outcomes with those of conventional therapy, as serious games provide an engaging, scalable method for delivering such interventions.

**Trial Registration:**

ClinicalTrials.gov NCT06596980; https://clinicaltrials.gov/study/NCT06596980

## Introduction

Specific learning disorder in reading (SLD reading), commonly named dyslexia, is a neurodevelopmental condition characterized by impairments in reading, writing, and phonological processing. SLD reading affects 5% to 10% of the population, with a relatively high prevalence in males. SLD reading is associated with deficits in phonological awareness, rapid naming, and auditory-verbal memory, which impair the ability to decode written language and recognize words fluently [1,2]. The recommended treatment always includes graphophonological interventions. Indeed, among the leading theories explaining SLD reading, the phonological deficit hypothesis posits that impaired representation, storage, or access to phonemes—the basic sound units of language—underlies reading difficulties [3].

A related theory, the temporal processing hypothesis, suggests that difficulties in perceiving rhythmic and prosodic features of speech, such as syllable stress and amplitude modulations, further exacerbate phonological and reading challenges [4,5]. Recent neurophysiological studies using electroencephalography have shown that children with SLD reading exhibit atypical low-frequency cortical encoding of speech, particularly in the delta and theta bands (1-8 Hz), which are critical for processing rhythmic and temporal aspects of language [6]. These findings align with genetic studies showing moderate but significant correlations between rhythm perception, language skills, and SLD reading, supporting the Atypical Rhythm Risk Hypothesis [7].

Rhythmic and musical interventions have emerged as promising tools for addressing these deficits. A scoping review examined the use of music interventions to improve reading skills in children with SLD reading [8]. The authors identified 12 studies that focused on how music interventions target auditory processing, phonological processing, and temporal processing. Rhythmic activities are the most common musical element used in interventions, primarily aimed at improving reading accuracy. These activities were designed to enhance phonological awareness, syllable segmentation, and word recognition. Music and rhythm engage neural networks similar to those involved in language processing, enhancing phonological awareness, auditory memory, and reading accuracy [9,10]. For example, rhythmic training improves the synchronization of neural oscillations with auditory stimuli, which is crucial for phonological segmentation and temporal prediction in speech [11,12]. These interventions are particularly effective because they target both the underlying cognitive deficits and the motivational barriers often faced by children with SLD reading.

Serious games, which combine educational objectives with engaging gameplay, offer a novel and scalable approach to SLD reading intervention [13]. These digital tools provide intensive, repetitive training in phonological and graphophonological skills, which are essential for reading development [14]. By incorporating adaptive difficulty levels, immediate feedback, and rewards, serious games maintain high levels of engagement and motivation, which are critical for children with SLD reading who may otherwise avoid conventional literacy activities [15,16]. Furthermore, these games can be accessed remotely, addressing disparities in access to specialized resources and enabling frequent, home-based practice [13,17]. To be effective, interventions for children with SLD reading must be intensive (5 times per week). However, achieving this intensity is not feasible in conventional in-person therapy, where speech therapists are typically limited to scheduling 1 to 2 sessions per week [18]. Therefore, using serious games appears to be a solution to support high-intensity training.

With this in mind, we developed Mila-Learn, a digital medical device in the form of a serious game designed for rhythmic training to improve reading skills in children aged 7 years to 11 years with SLD reading [17]. It includes a series of music-based games focusing on rhythm repetition, synchronization, and rhythm completion, which are embedded in a story where the child (here, the player) is the hero. Between October 2021 and March 2023, we conducted a randomized placebo-controlled trial with 154 children with SLD reading who played with Mila-Learn or with a placebo game for 2 months. The results revealed improvements in reading accuracy and speed, supporting the ability of Mila-Learn to improve reading in children with SLD reading [19].

However, recommendations for best practices in the treatment of written language disorders in children [1] include 3 different modes of intervention for patients with SLD reading: (1) curative treatment targets underlying cognitive deficits (eg, phonological or visual-attentional deficits) and the various processes involved in written word identification (eg, graphophonological conversion, phonological decoding, orthographic memory, orthographic recoding), (2) adaptive treatment aiming to strengthen the reader’s natural compensatory strategies (eg, development of lexical orthographic memory), and (3) compensatory treatment to reduce written language disorders by replacing deficient cognitive functions (eg, using digital aids). These intervention modes are not mutually exclusive and should be combined or alternated according to a treatment plan tailored to each patient. However, whenever possible, curative treatment should be the first intervention used with the patient. Curative treatment needs to combine phonological training with graphophonological training, as phonological training alone results in limited improvement in reading skills [1].

The publication of these recommendations led to modifications in the Mila-Learn game to better address the various aspects of curative treatment. In addition to the rhythmic games, we computed and added games focusing on written word identification. The new medical device, named Poppins, thus combines rhythmic tasks from Mila-Learn with new written language training tasks. In this article, we first describe the language games added to Poppins. We describe a single-arm study in which we focus on the impact of Poppins on the reading and phonological skills of children with SLD reading. As an exploratory analysis, we then compared the effects of Poppins with those of the medical device Mila-Learn [19] on the reading and phonological skills of children with SLD reading.

## Methods

### Device Description

Poppins is an app available on tablets and smartphones (iOS and Android) in which the child is led to carry out different activities divided into 2 categories: language activities and rhythm activities. Game data are stored anonymously on secure servers that comply with medical data protection regulations.

Overall, Poppins contains a number of different short activities, allowing for a variety of activities within a single session. An activity lasts an average of 1.40 minutes, to not demand too much attention from the child [20,21]. This structure of short activities ensures a fluid, coherent, and continuous experience for the user. To offer an experience suitable for as many children as possible (from 7 years to 11 years old, gamers and nongamers, with a wide range of tastes), the visual and narrative aesthetics of Poppins are aligned with mainstream video game standards such as Rayman and Mario games, games for all audiences.

**Figure 1:**
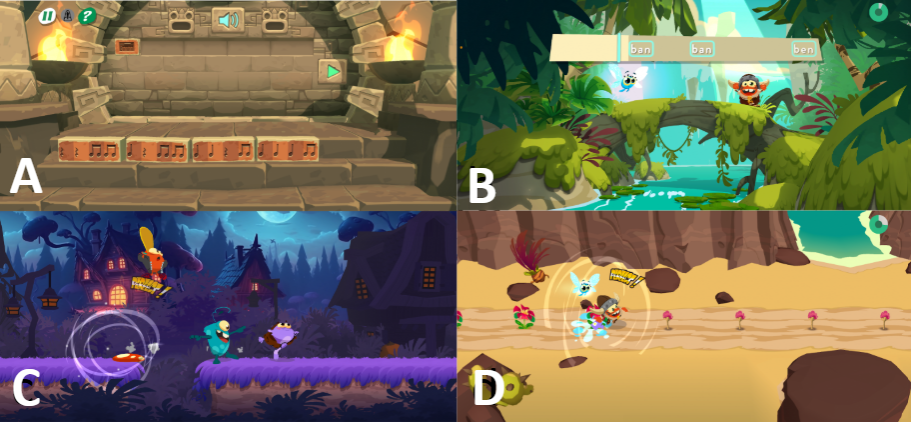
Screenshots of rhythm tasks in the Poppins medical device: (A) In the Temple of Music, the child must recreate the rhythm of a melody using predefined rhythmic blocks; (B) in the King Song, the child must sing the displayed syllables in rhythm; (C) in the Beat Jumper, the child must make their character jump in rhythm by shaking the tablet; and (D) in Pop'n Run, the child must keep the rhythm by tapping the screen with their finger.

Rhythmic activities (Figure 1) consist of rhythmic activities that were previously described [17]. To quickly summarize, all the tasks were designed to work on rhythm, as rhythm appears to be directly related to reading skills, whereas melody is not [22]. However, each task requires the mobilization of other skills, such as attention, inhibition, working memory, and motor skills, which are also often impaired in children with SLD reading [23].

In March 2022, Collège Français d’Orthophonie (French College of Speech Therapy [CFO]) [1] published its recommendations for best practices in written language. These best practice recommendations were developed using the formal consensus recommendation method described in the scientific rationale published by the Haute Autorité de Santé [24] and include a section on the management of SLD reading. These recommendations reiterate the principle of the phonological hypothesis and the deficit in temporal processing of auditory signals. The approach of rhythm-based intervention is presented and supported by recommendations, which advise a “curative” intervention as a first-line treatment, meaning that it aims to rehabilitate underlying processes (such as rhythm processing) in the context of SLD reading.

However, this curative intervention includes 2 components: the rehabilitation of underlying processes and direct work on graphophonological conversion (ie, the link between graphemes [letters] and phonemes [sounds]). The CFO’s recommendations [1] also provide guidelines for implementing graphophonological conversion training. Following the publication of these recommendations, the game Mila-Learn was revised to include exercises targeting graphophonological conversion, which we will refer to as the language games module, in addition to the rhythmics tasks already developed. The 20-minute training time is evenly divided between written language exercises and rhythmic tasks. Parents are advised to aim for 5 play sessions per week. In the game, specific rewards appear starting from the 3rd session to encourage the child to complete at least 3 sessions per week, with additional rewards unlocked upon reaching the fifth session. The child’s play time was monitored directly by the app. Once the child had played for 20 minutes, the game automatically stopped. The play time data were transmitted directly to Poppins’ company using the internet. Parents were encouraged to ensure that the tablet was connected to the internet after each session that was played by the child. If the child did not play for a week, notifications were sent by the app: one on Monday to remind the child to play and another on Saturday to encourage play over the weekend. In addition, parents received an email on Wednesday, reminding them that their child should play Poppins.

**Figure 2:**
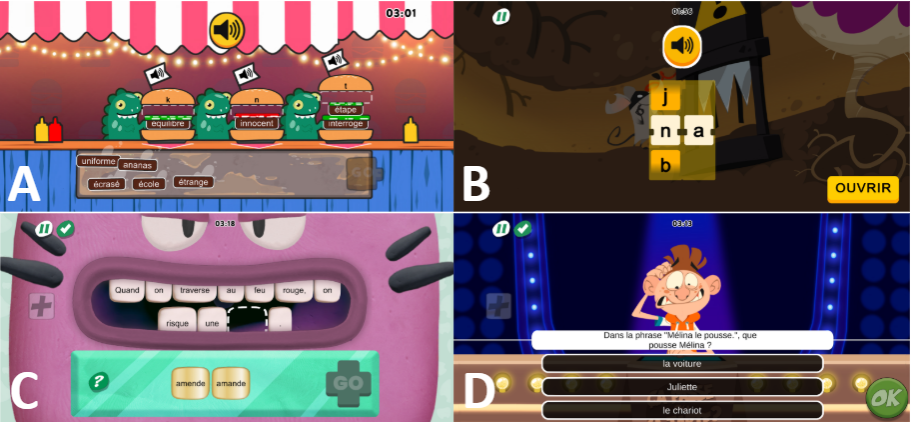
Screenshots of language tasks in Poppins medical device: (A) In Dino-Burger, the child must categorize words on the basis of the sounds they contain; (B) in Veggie Crok, the child must reconstruct a syllable or a word; (C) in Supersmile, the child must choose the correct word to complete the sentence; and (D) in Ca. passe ou ça tarte (meaning “Pass or Smash”), the child must answer a question to demonstrate sentence comprehension.

All written language games (Figure 2) are designed to present the child with the grapheme linked to its phoneme and consist of real words selected on the basis of their frequency (using the Manulex-infra database [25]), orthographic complexity (verified using the Eole database [26]), and length. Words are presented to the child on the basis of their infralexical characteristics. The difficulty level is continuously adapted to the child on the basis of their responses in the game: Depending on the number of errors in each exercise, the complexity level of the words is adjusted, and the child’s progress in the game is tailored. The games are designed to maximize errorless learning, and immediate feedback is provided if the child makes a mistake. For example, in the word search game, the target words are displayed to the child, minimizing the risk of spelling errors.

The written language games are divided into 4 areas: one focused on phonology (“I listen”), one focused on reading (“I read”), one focused on transcription (“I write”), and the last focused on comprehension (“I understand”). These areas always work in pairs to limit the number of different tasks (Recommendation 2.8) [1]. Additionally, these areas alternate on the basis of the child’s progress, allowing for the coordination of reading tasks with transcription tasks (Recommendation 2.13) [1].

### Study Protocol

**Figure 3:**
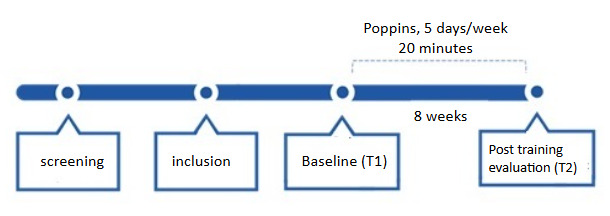
Description of the protocol for a single-arm study involving dyslexic children aged 7 years to 11 years.

The study design is a single-arm trial with no active control group examining the effect of the Poppins digital medical device for children with SLD reading (Figure 3). Each participant included in the study followed the training protocol, which consists of 5 training sessions per week on Poppins. Each session lasts approximately 20 minutes. After an assessment of their reading and phonological skills, all the children trained for 8 weeks with the Poppins medical device. Their skills were reassessed after the training period.

### Participants

We included children aged between 7 years and 11 years with a diagnosis of SLD reading according to the Diagnostic and Statistical Manual of Mental Disorders, Fifth Edition (DSM-V) criteria, as confirmed by the recruiting center’s physicians. They had to be native French speakers or have been schooled in France for at least 3 years. The children were not allowed to have previously played Mila-Learn or Poppins. Children with autism, intellectual disabilities, or epilepsy were also excluded.

The sample size determination was based on the main objective and performed in G.Power 3.1.9.7.

The null (H0) and alternative (H1) hypotheses are defined as follows: H0: µD=0 and H1: µD≠0, where D=EVAL2MT2-EVAL2MT1 (defined earlier as the primary end point).

A 1-sample bilateral *t* test, assuming a dz effect size of 0.5, a 5% type I error, and 80% power, determined that the study needs 34 participants. To account for a 10% dropout rate (34/0.9), 38 participants were needed.

### Outcomes

The primary variable was reading ability as assessed using the Eval2M task, which involves reading a list of words within a 2-minute time frame and produces 2 scores (speed and accuracy; Evaleo 6-15) [27].

The secondary variables included phonological skills, measured through a phoneme deletion task (BALE) [28] and a phonemic discrimination task (Evaleo 6-15) [27]. Additionally, reading skills were assessed in terms of speed and accuracy using the nonsensical text “Evalouette” [27], as well as in terms of speed, accuracy, and comprehension using texts from Evaleo 6-15 [27]. We also assessed parental stress with the shortened version of the Parenting Stress Index [29] and children’s quality of life with the quality-of-life questionnaire of the PedsQL 4.0 Generic Core Scales child report [30] 8 weeks after the start of the training. The PedsQL 4.0 Generic Core Scales is a widely used and validated 23-item instrument measuring health-related quality of life in children and adolescents. For this study, the child self-report version was used. The Parenting Stress Index-Short Form is a 36-item parent self-report questionnaire. It efficiently measures parental stress across 3 domains: Parental Distress, Parent-Child Dysfunctional Interaction, and Difficult Child. Finally, all adverse events reported by the participants were recorded to evaluate the safety of Poppins.

### Ethical Considerations

The study protocol was approved by the local ethics committee (Comité de protection des personnes Ile de France; approval number 24.00890.000301) and national regulatory agencies (Agence nationale de sécurité du médicament et des produits de santé and Commission nationale de l'informatique et des libertés; approval number 2024-A00432-45).

Prior to providing consent, the patient and their parents received a complete and comprehensive explanation of the study, including the study rationale, the procedures, the benefits and risks, that participation was voluntary, and that the participant could withdraw from the study at any time without any negative consequences.

Written informed consent was obtained from the participant or legal guardian in accordance with local practice and regulations prior to any study assessment or test being conducted. The parents provided their signature at the end of the consent form, and the delegated site team member countersigned the form. Consent was obtained via a wet-ink signature. A copy of the fully executed informed consent form was provided to the participants and their parents (either a paper version or electronically sent via email), and a copy was retained by the site in a secure area with restricted access.

Given the patient’s condition (specific learning disability with reading or written expression deficit) and his or her age, thus potentially being unable to provide written consent, the child provided verbal assent to participate in the trial. The investigator read the content of the consent form during the information visit, clearly stating that the child was free to decline his or her participation at any moment. The consent form was adapted to an appropriate language to facilitate the child’s understanding. Once the child verbally agreed to participate in the trial, the investigator signed the consent form on their behalf and registered it in the electronic case report form.

Information and inclusion visits could be carried out remotely or in person at the discretion of the parents or the investigator if he or she considered it necessary to assess that the child was not subjected to undue pressure.

Identifiable participant details (confidential details, including name and date of birth) were held in a database separated from the research database after receiving participants’ consent. The research database never holds personal identifiable information. Only clinical sites had access to this information.

The study was registered in ClinicalTrials.gov retrospectively on September 11, 2024, after it started on June 28, 2024.

### Statistical Analysis

Statistical analyses were carried out using R Version 4.2.2. First, we aimed to assess the delta before and after training with Poppins. In line with our predefined statistical analysis plan, the evaluation of absolute changes in skills between the baseline assessment (T1) and final assessment (T2) was conducted using a mixed linear regression with the following formula: score ~ time (T2 vs T1) + (1|Subject), with a *t* test of the time effect (lme4 package). The participant was used as a random intercept to account for individual variability. *P* values and 95% CIs were obtained using bootstrap resampling (boot package; 10,000 replications).

In a preceding clinical trial [19], 8 weeks of training with Mila-Learn was compared with 8 weeks of training with a placebo game. Our second exploratory analysis aimed to compare the scores of the children exposed to Poppins (this study) with the scores of the children exposed to the former version of the digital medical device Mila-Learn (previous study). To compare the results, the former version of the digital medical device will be compared when the results are significantly different from those of the placebo in search of superiority and difference descriptions. The comparison of change scores (T2-T1) between groups was performed using the Welch *t* test. Missing data were handled using a complete-case analysis.

## Results

### Study Follow-Up

**Figure 4:**
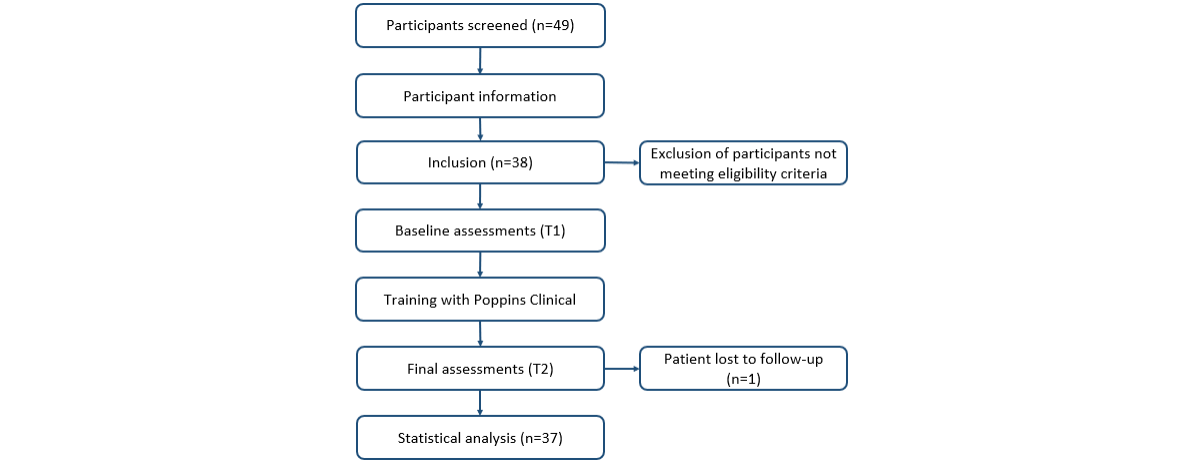
Flowchart of patient recruitment and study follow-up.

The study started in June 2024 and ended in February 2025, and 49 patients were screened for this study. The inclusion criteria were not met by 11 patients. A total of 38 patients gave their informed written consent to participate in the study and were subsequently enrolled (see Figure 4). One patient was lost to follow-up. Consequently, 37 patients were included in the analysis. All patients followed the training during the study.

### Participants

The characteristics of the participants enrolled are described in Table 1.

**Table 1:** Description of the participants recruited for the study on the basis of their grade level.

Characteristics			2nd grade (n=6)		3rd grade (n=13)		4th grade (n=14)		5th grade (n=5)
Age (years), mean (SD)			8.21 (0.47)		8.94 (0.42)		9.91 (0.62)		10.88 (0.46)
Sex, n									
	Female	1		8		4		0	
	Male	5		5		10		5	
Speech therapy, n (%)			2 (33)		11 (85)		9 (64)		2 (40)
ADHD^a^, n (%)			4 (67)		4 (31)		3 (21)		2 (40)
Language disorder, n (%)			0 (0)		1 (8)		0 (0)		0 (0)
Dyspraxia, n (%)			1 (17)		2 (15)		2 (14)		0 (0)
Dyscalculia, n (%)			1 (17)		3 (23)		1 (7)		0 (0)

^a^ADHD: attention-deficit/hyperactivity disorder.

**Figure 5:**
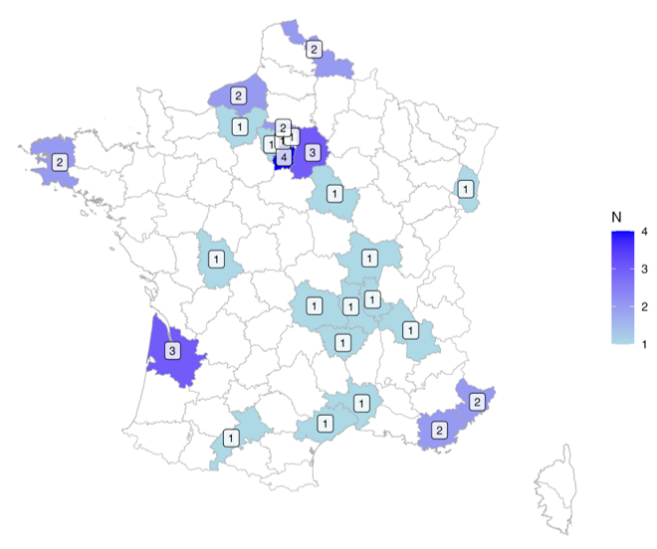
Geographical distribution of children included in the study across France.

Among the 38 participants, 20 had a medical history. The highest level of education attained by the parents was controlled. Among the 38 families, none did not have a high school diploma (Bac), 18 (47%) had an education level ranging from Bac to Bac +3, and 20 (53%) had an education level equal to or higher than Bac +4. The mean total time played by the children was 653 (SD 202) minutes. The distribution of children in France is shown in Figure 5. No adverse events were reported during the study.

### Outcomes

The primary end point was absolute improvement in reading skills, specifically through the lens of word reading capability, using the EVAL2M subtest from EVALEO 6-15 [27] after an 8-week training regimen (T2). The secondary end point was improvement in reading skills in terms of accuracy, speed and comprehension, and phonological skills (phoneme deletion and phonological discrimination). The scores at T1 and T2 are presented in Table 2.

**Table 2:** Comparison of participants’ scores on the proposed tasks at pretest (T1) and posttest (T2).

Tasks		T2 (n=37), mean (SD)	T1 (n=38), mean (SD)	Difference (95% CI)	*P* value
**Words read in 2 minutes (EVAL2M)**					
	Words correctly read	101.49 (33.86)	88.39 (34.00)	11.46 (8.75 to 14.25)	<.001
	Words read	110.46 (32.43)	98.50 (32.54)	10.26 (6.77 to 13.85)	<.001
**Phoneme deletion (BALE)**					
	Total score	16.58 (3.38)	13.54 (4.51)	2.87 (1.64 to 4.09)	<.001
	Total time (seconds)	205.19 (101.37)	213.14 (102.56)	–5.37 (–31.97 to 21)	.69
**Text with no meaning (Evalouette)**					
	Words read	105.59 (42.94)	93.21 (38.76)	10.95 (4.87 to 17.19)	<.001
	Words correctly read	94.49 (42.80)	79.76 (38.92)	13.25 (7.78 to 18.86)	<.001
**Phonological discrimination (Evaleo 6-15)**					
	Total score	21.84 (3.95)	20.21 (3.86)	1.61 (0.2 to 3.06)	.03
**Text comprehension 4th and 5th grades**					
	Number of reading errors	5.50 (5.73)	4.50 (4.88)	1.00 (–0.72 to 2.67)	.25
	Reading time (seconds)	97.76 (41.51)	85.29 (37.46)	12.46 (4.96 to 19.76)	.001
	Comprehension score	15.45 (1.50)	4.75 (1.12)	0.70 (0.06 to 1.32)	.03

The results indicate that participants correctly read significantly more words at T2 than at T1 (*P*<.001). On average, children read 11.46 more words correctly at T2 than at T1. The participants using Poppins also showed improvements in reading speed, with more words read at T2. The difference in mean improvement (+10.26 words read between T1 and T2) reached statistical significance (*P*<.001).

Children significantly improved their performance between T1 and T2 for reading speed and fluency (*P*<.001) and phonological tasks. No significant improvement between T1 and T2 was found in the parental stress questionnaire (*P*=.15) or in the child’s quality of life questionnaire (*P*=.92).

Text comprehension was assessed using the paragraph reading task from EVALEO 6-15 [27]. This assessment varies depending on grade level, as the texts provided differ between the 2nd and 3rd grades and the 4th and 5th grades. The results were therefore analyzed by grade level, 2nd/3rd vs 4th/5th. No significant improvement in reading time (*P*=.66), the number of reading errors (*P*=.052), or the comprehension score (*P*=.86) was found in the text comprehension task for the 2nd/3rd graders. A significant improvement in the comprehension score (*P*=.03) was found for the 4th/5th graders, as the reading time increased (*P*=.001).

Finally, we conducted an exploratory analysis comparing the group who played Poppins and the group who played Mila-Learn in a previous study [19] in terms of absolute improvement in the number of words read and number of words correctly read using the subtest EVAL2M from the Evaleo 6-15 [27] and in terms of absolute improvement in the score of the phoneme deletion task from the BALE [28] after an 8-week training regimen (T2). The change in each group was quantified as the difference between the posttraining mean score at T2 and the pretraining baseline mean score. The results are summarized in Table 3.

**Table 3:** Improvement in scores between posttest (T2) and pretest (T1) for children using Poppins compared with the improvement in scores for children using Mila-Learn from the previous study.

Tasks		Poppins (N=38), mean (SD)	Mila-Learn (N=75), mean (SD)	Difference (95% CI)	Cohen *d*	*P* value
**Number of words correctly read (EVAL2M)**						
	T2-T1	11.41 (8.44)	10.63 (12.51)	0.77 (–3.31 to 4.85)	0.07	.70
	T1	88.39 (34.00)	89.32 (30.39)	–0.92 (–14.05 to 12.20)	–0.03	.90
**Number of words read (EVAL2M)**						
	T2-T1	10.16 (10.86)	11.25 (13.43)	–1.09 (–5.89 to 3.72)	–0.09	.70
	T1	98.50 (32.54)	102.72 (27.07)	–4.22 (–16.54 to 8.10)	–0.15	.50
**Phoneme deletion score (BALE)**						
	T2-T1	2.75 (3.67)	0.90 (3.94)	1.85 (0.31 to 3.40)	0.48	.02
	T1	13.54 (4.51)	12.38 (5.22)	1.17 (–0.75 to 3.08)	0.2	.20

The progression of children’s scores was similar between Mila-Learn and Poppins for speed (number of words read) and reading fluency (number of words read correctly). However, the phonological skills of the children in the Poppins game group were better than those of the children in the Mila-Learn game group.

## Discussion

### Principal Findings

In this study, we evaluated the impact of 8 weeks of training with Poppins on the reading performance of children aged 7 years to 11 years with SLD reading. We also assessed the phonological skills of the children and compared the results of the group who used the medical app Poppins with those of a group who used a former version of the digital medical device (named Mila-Learn) in a prior study. SLDs manifest as marked deficits in reading fluency and accuracy and are typically characterized by slow, effortful reading and substantial inaccuracies [2]. Reading accuracy precedes the development of reading speed, with significant improvements in speed occurring once a foundational accuracy level is established [31]. Therefore, both reading accuracy and speed are crucial metrics for assessing reading proficiency.

First, Poppins appears to be a safe device, as no adverse events were reported during the study. No device malfunction could lead to a serious adverse event, and no serious breaches were reported.

We explored clinical performance by assessing children after 8 weeks of training with Poppins. With respect to reading accuracy, the children improved their scores in nearly all reading tasks after 8 weeks of training with Poppins. Children showed improvement in the primary end point, with the number of correctly read words increasing significantly (*P*<.001), by 11 words. For comparison, children in the placebo group in the previous study [19] improved their reading by 5 words at the second evaluation, 8 weeks later. This improvement was also observed in other reading tasks, such as reading nonsense text (Evalouette; *P*<.001). Word reading is a measure used in clinical and research settings, serving as a proxy for reading proficiency in day-to-day life.

The impact of Poppins on reading speed has also been investigated. Reading fluency is recognized as a key marker of SLD reading across different languages and is a persistent symptom of SLD reading, even when basic decoding skills have been acquired [32]. In our study, reading speed was reported by counting the number of words read, which improved at T2 (*P*<.001).

Phonological awareness is an important capacity when exploring reading. Indeed, phonological deficit is one of the most common causes of SLD reading [3,33] and results, among others, in phonological awareness difficulties. In our study, children's scores improved on both the discrimination and the phoneme deletion tasks (*P*=.03 and *P*<.001, respectively). The children did not show significant improvement in speed in the deletion task (*P*=.69). Assessing phonological skills in children with SLD reading typically involves evaluating both accuracy and speed, as these dimensions provide a comprehensive understanding of their phonological processing abilities. Many children with SLD reading exhibit deficits in phonological processing speed, meaning that they process phonological information more slowly than their peers do [34]. Speed is often assessed using tasks such as rapid automated naming, which measures how quickly a child can name familiar items (eg, letters, colors, or objects). In future research, it could be valuable to include such tasks in the assessment battery to better capture improvements in phonological processing speed.

We evaluated the reading comprehension of children after an 8-week intervention with Poppins. The assessment was conducted using the paragraph comprehension task of EVALEO 6-15 [27]. This task involves 2 texts to avoid retest effects: one for the initial evaluation and another for the follow-up evaluation. The texts are adapted to the children’s grade level, with children in 2nd and 3rd grades reading shorter texts consisting of only a few sentences, whereas children in 4th and 5th grades read a full text composed of 3 paragraphs, each containing 5 to 6 sentences. Reading comprehension was assessed by asking questions about the text. The results revealed no significant effect of the training on the comprehension scores of children in the 2nd and 3rd grades. However, the comprehension scores of older children in grades 4 and 5 improved, as evidenced by the analyses (*P*=.03). The observed difference in results between 2nd/3rd grade and 4th/5th grade students likely stems from the texts used. The texts for 4th/5th grade students are significantly longer than those for 2nd/3rd grade students, leading to increased working memory load. Additionally, the comprehension of 4th/5th grade students is assessed with a greater number of questions than that of 2nd/3rd grade students (7 questions vs 4 questions), allowing for a more detailed evaluation of their text comprehension abilities. We also observed that children read more slowly at T2 than at T1 (+12.46 seconds; *P*=.01). This suggests that children may have slowed their reading speed to ensure better comprehension of the text, which is a common and effective strategy among children with SLD reading [35].

### Comparison With the Former Version Mila-Learn

Finally, we compared the evolution of reading accuracy and reading speed scores obtained from the 2-minute word reading task (EVAL2M) between children who used Poppins and those who used Mila-Learn. No significant differences were found between the 2 groups, suggesting that the new version of the device, Poppins, remains effective. We also examined the impact of Poppins on phonological skills, as measured by the phoneme deletion task, compared with the impact of Mila-Learn. Children who used Poppins improved their scores by 1.85 points more than those who used Mila-Learn did (*P*=.02). In the previous Poppins-01 study, only children in the 2nd/3rd grades demonstrated significantly greater improvement in the Mila-Learn group than in the placebo group. These results support the hypothesis that Poppins enhances children’s phonological skills more effectively than the previous version, Mila-Learn. Indeed, several studies have independently demonstrated the benefits of rhythmic training, as well as phonemic and graphophonological training, in improving phonological skills in children with dyslexia [36,37]. These different approaches have shown substantial effects, which are generally comparable across interventions. Thomson et al [36] suggested that combining rhythmic training with graphophonological training may be particularly effective for enhancing phonological skills in children with dyslexia.

Among the study population, 9 children played with Poppins for less than 500 minutes, representing 27% of the population, whereas 36% of the children in the previous study using the Mila-Learn game did not reach the 500-minute play threshold. The median playing time was 752.50 minutes, with the majority of the children playing for more than 500 minutes (Q1=524.00 minutes, Q3=809.50 minutes). The mean global playing time was 653 minutes, which is comparable to the playing time reported in a previous study with Mila-Learn (global playing time=660 minutes). This suggests that the addition of language exercises did not negatively impact adherence to the treatment.

Maintaining treatment adherence makes Poppins a relevant tool for health care professionals to enhance patient care by enabling home-based training. Moreover, serious games offer affordable and accessible solutions, providing support when health care services are limited or in the context of remote follow-up. Treatment adherence is further supported by the game’s engaging and playful elements [38].

### Limitations

Despite these promising results, our study has several major limitations. First, we did not include a control group that followed their treatment as usual, which makes it difficult to attribute the observed improvements to the use of Poppins rather than to natural progression for the majority of our results. To address this, we used data from a previous study [19] as a comparison, but this approach may affect the validity of the comparison. To minimize bias, we conducted recruitment in the same way for both studies and used the same inclusion criteria, except for the speech therapy sessions. Effectively, children in our current study were allowed to continue their usual treatment. Among the 38 recruited children, 24 (63%) were receiving speech therapy. In contrast, the children in the previous Poppins-01 study did not have speech therapy sessions. This difference may have introduced bias, as children in the Poppins group could have received additional training than those in the Mila-Learn group did, and we did not monitor the exact number of speech therapy sessions attended by the children during their participation in the study. However, since a large portion of the children were recruited between June and July, many did not regularly attend speech therapy during this period, as July and August are holiday months in France. Finally, the groups also differed in terms of age and gender distribution, with a greater percentage of children in 5th grade (CM2) in the Mila-Learn group (23/75, 30% for Mila-Learn vs 5/38, 13% for Poppins) and a greater percentage of girls who played Mila-Learn (39/75, 52% for Mila-Learn vs 13/38, 34% for Poppins) [19].

### Conclusion

In conclusion, despite several limitations, the study revealed that Poppins appears to be a promising tool, with participants showing significant improvements in reading fluency as well as phonological skills. The improvements in reading skills were comparable to those seen with the previous version of the device, Mila-Learn, but Poppins might have a greater impact on phonological skills. The improvement in reading seems to have lightened the load on working memory, allowing children to better understand long texts. Moreover, no adverse events were reported, and the device was found to be safe and well tolerated.

## Abbreviations

Bac: high school diploma
CFO: Collège Français d’Orthophonie
DSM-V: Diagnostic and Statistical Manual of Mental Disorders, Fifth Edition
SLD-reading: specific learning disorder with reading impairment

## Data Availability

The data sets generated or analyzed during this study are available from the corresponding author upon reasonable request.
