# Correction: Children’s Improvement After Language and Rhythm Training With the Digital Medical Device Poppins for Dyslexia: Single-Arm Intervention Study

**DOI:** 10.2196/93737

**Published:** 2026-03-12

**Authors:** Charline Grossard, Melanie Descamps, Hugues Pellerin, François Vonthron, David Cohen

**Affiliations:** 1 Department of Child and Adolescent Psychiatry Pitié-Salpêtrière Hospital Paris France; 2 Poppins Paris France

In “Children’s Improvement After Language and Rhythm Training With the Digital Medical Device Poppins for Dyslexia: Single-Arm Intervention Study” [1], the authors made one correction.

In Table 2, the score for the subtest comprehension at T2 has been revised from:

15.45

To:

5.45

Table 2 has been revised to the following:

**Table 2:** Comparison of participants’ scores on the proposed tasks at pretest (T1) and posttest (T2).

Tasks		T2 (n=37), mean (SD)	T1 (n=38), mean (SD)	Difference (95% CI)	*P* value
**Words read in 2 minutes (EVAL2M)**					
	Words correctly read	101.49 (33.86)	88.39 (34.00)	11.46 (8.75 to 14.25)	<.001
	Words read	110.46 (32.43)	98.50 (32.54)	10.26 (6.77 to 13.85)	<.001
**Phoneme deletion (BALE)**					
	Total score	16.58 (3.38)	13.54 (4.51)	2.87 (1.64 to 4.09)	<.001
	Total time (seconds)	205.19 (101.37)	213.14 (102.56)	–5.37 (–31.97 to 21)	.69
**Text with no meaning (Evalouette)**					
	Words read	105.59 (42.94)	93.21 (38.76)	10.95 (4.87 to 17.19)	<.001
	Words correctly read	94.49 (42.80)	79.76 (38.92)	13.25 (7.78 to 18.86)	<.001
**Phonological discrimination (Evaleo 6-15)**					
	Total score	21.84 (3.95)	20.21 (3.86)	1.61 (0.2 to 3.06)	.03
**Text comprehension 4th and 5th grades**					
	Number of reading errors	5.50 (5.73)	4.50 (4.88)	1.00 (–0.72 to 2.67)	.25
	Reading time (seconds)	97.76 (41.51)	85.29 (37.46)	12.46 (4.96 to 19.76)	.001
	Comprehension score	*5.45* (1.50)	4.75 (1.12)	0.70 (0.06 to 1.32)	.03

The correction will appear in the online version of the paper on the JMIR Publications website, together with the publication of this correction notice. Because this was made after submission to PubMed, PubMed Central, and other full-text repositories, the corrected article has also been resubmitted to those repositories.
